# Modulation of ER Stress via cAMP Signaling: Protective Role of Rolipram in Paracetamol-Induced Liver Injury

**DOI:** 10.1021/acs.chemrestox.6c00126

**Published:** 2026-04-21

**Authors:** Saziye Sezin Palabiyik-Yucelik, Nagihan Demirtas, Nevra Aydemir Celep, Ayse Bozkurt, Zekai Halici, Elif Cadirci

**Affiliations:** † Faculty of Pharmacy, Department of Pharmaceutical Toxicology, 37139 Ondokuz Mayıs University, Samsun 55400, Turkey; ‡ Faculty of Medicine, Department of Medical Pharmacology, 37503 Atatürk University, Erzurum 25240, Turkey; § Faculty of Pharmacy, Department of Pharmaceutical Toxicology, Atatürk University, Erzurum 25240, Turkey; ∥ Department of Histology and Embryology, 570908 Atatürk University, Faculty of Veterinary Medicine, Erzurum 25240, Turkey; ⊥ Faculty of Pharmacy, Department of Pharmacology, Van Yüzüncü Yıl University, Van 65080, Turkey

## Abstract

This investigation sought to assess the potential protective function of the phosphodiesterase-4 (PDE) enzyme inhibitor rolipram (ROL) against paracetamol (PARA) induced liver damage, as well as any potential underlying mechanism through modulation of endoplasmic reticulum (ER) stress via cAMP signaling. Fifty-six rats were randomly divided into 7 groups (*n* = 8). Following 24 h of fasting, animals received three different dosages of ROL (1.25, 2.5, and 5 mg/kg, i.p.) or *N*-acetylcysteine (140 mg/kg, orally). One hour later, 2 g/kg PARA orally was administered to induce hepatotoxicity. The ELISA method was used to evaluate GSH, PDE4D, and cAMP levels in liver tissue, as well as PDE4D and cAMP levels in serum. In addition, serum levels of liver injury biomarkers, including ALT and AST, were measured. GRP78, IRE1, and CHOP mRNA expressions in tissue samples were assessed using the Real Time PCR technique. The liver tissue’s histopathological parameters, including necrosis, bleeding, and mononuclear cell infiltration, were evaluated. The PARA group had higher serum and tissue PDE4 levels, lower cAMP and GSH levels, higher ALT and AST levels, and higher expressions of the ER stress markers GRP78, IRE1, and CHOP mRNA. Histopathological evaluation also revealed severe histopathological damage with PARA toxicity. These changes improved with dose dependent increase of ROL dose. It was evaluated that the protective effect of PDE4 enzyme inhibition on liver injury caused by PARA toxicity could be regulated by intracellular secondary communication signals and ER stress inhibition. These findings also suggested that these pathways might be studied in relation to other liver injuries.

## Graphical abstract

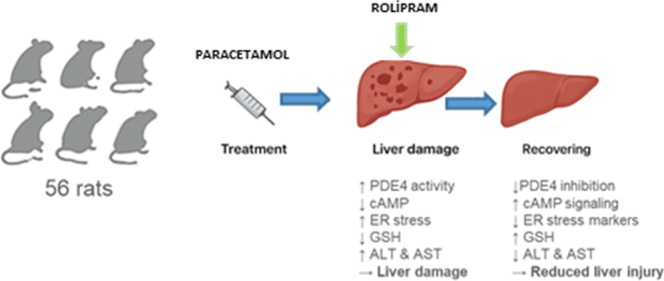

## 1 Introduction

Paracetamol (PARA) is one of the most commonly used over-the-counter medication that is considered safe at therapeutic doses. However, paracetamol overdose is a well-established cause of drug-induced hepatotoxicity and represents a leading cause of acute liver failure in many countries. Currently, *N*-acetylcysteine (NAC) is the standard treatment for PARA toxicity but its therapeutic window is narrow and its efficacy diminishes when administered beyond the early stages of intoxication. Therefore, studying models of drug-induced liver injury is crucial for identifying agents with preventive or therapeutic potential.^1^ Moreover, the use of such models is essential for elucidating the molecular mechanisms underlying hepatotoxicity, which in turn can inform the development of targeted treatment strategies.^2^

The first prototype inhibitor of the phosphodiesterase (PDE)-4 enzyme, rolipram (ROL), originally identified for its antidepressant properties.^3^ Although it is not currently used in clinical practice, ROL remains a valuable experimental tool for investigating the underlying molecular mechanisms of PDE4-related pathologies.^4,5^ Cyclic adenosine monophosphate (cAMP) is degraded by PDEs, which thereby regulate its levels across various subcellular compartments.^6^ cAMP is a well-studied molecule with critical roles in liver physiology and pathology.^7–10^ Increased intracellular cAMP levels alter lipid metabolism, energy homeostasis, immunological responses, inflammation, cell proliferation, and motility by activating downstream effector molecules like the classical protein kinase A (PKA) and exchange proteins that are directly activated by cAMP.^7^ The cAMP/PKA signaling pathway can also interfere with hepatic metabolism by reducing cytochrome P450 2E1 ^11^.

Endoplasmic reticulum stress (ERS) is a signaling pathway involved in both physiological and pathological processes, and it plays a critical role in elucidating the interaction between cell death pathways and molecular mechanisms associated with toxicant exposure. Investigating these interactions may provide insights into preventing toxicant-induced organ damage and developing novel therapeutic strategies.^2^ Both in vivo and in vitro studies have reported that PARA can induce ERS and initiate a cascade of signaling events in hepatocytes, suggesting that ERS may play a significant role in PARA-induced liver injury.^12–14^ However, the precise mechanisms by which PARA induces ERS remain poorly understood.^12^ Moreover, the endogenous regulatory mechanisms responsible for mitigating ERS during PARA-induced liver injury are largely unknown. Supporting our hypothesis, it is proposed that inhibition of PDE4 increases intracellular cAMP concentrations, which in turn activates Nrf2. Activated Nrf2 translocate to the nucleus, where it enhances the expression of HO-1, thereby promoting the clearance of reactive oxygen species (ROS) that trigger ERS.^15^ Notably, a study by Rodriguez et al. (2019) demonstrated that ROL through PDE4 inhibition, attenuates ERS in alcoholic liver injury models.^16^

Although the protective effects of various xenobiotic, including drugs, have been evaluated through different mechanisms in liver injury induced by PARA toxicity, to our knowledge, there is no study assessing the potential effect of the PDE4 inhibitor ROL in this context. The role of cAMP has also been investigated in different types of liver injury and PARA-induced liver injury.^8–10,17^ While the ERS pathway proposed for evaluation in this study has been previously investigated in the context of PARA toxicity^14,18^ no study in the literature has simultaneously assessed the potential protective role of ROL, a PDE4 inhibitor, in this pathway. In particular, the relationship between PDE4 inhibition, increased cAMP levels, and ERS-related biomarkers in PARA-induced liver injury has not been fully elucidated. Therefore, the aim of the present study is to investigate the potential protective role of PDE4 enzyme inhibition, in acute liver injury induced by PARA toxicity, and to elucidate the potential mechanism by which elevated cAMP levels may modulate ERS. This study also seeks to uncover possible pathways relevant to drug- and xenobiotic-induced liver injuries.

## 2 Materials and Methods

### 2.1 Animals

Fifty-six male Albino Wistar rats (weighing 240–270 g) were obtained from the ATADEM Laboratory Animal Research Center in Erzurum, Turkey. During the experiment, animals were provided ad libitum access to standard pellet feed and water. Prior to the experiments, all animals were acclimatized under regular laboratory conditions (22 °C room temperature and a 12:12 h light/dark cycle). Every experimental technique was conducted in compliance with the European Union Directive 2010/63/EU. The local ethics committee for animal experiments at Atatürk University granted ethical approval (approval no: 172; Date: September 28, 2023; Document No: E–75296309-050.01.04-2300313533).

### 2.2 Chemicals

We purchased paracetamol Galenik (İzmir, Turkey), Rolipram (R0110) was purchased from TCI (Zwijndrecht, Belgium), NAC Sigma-Aldrich (St. Louis, USA). The ELISA kits used were cAMP (Catalog #201110299) and cAMP Specific (PDE4D) (Catalog #201116385), both supplied by SunRedBio (Shanghai, China).

### 2.3 Study Design

A total of 56 Albino Wistar rats were randomly assigned to seven groups (*n* = 8), as outlined in Table 1 and summarized in Figure 1.

**Table 1:** Experimental Design of Study^a^

groups	number of animals	treatment	dose/volume
I	8	Healthy	1 mL ROL vehicle +2 mL PARA vehicle
II	8	ROL	5 mg/kg ROL +2 mL PARA vehicle
III	8	PARA	1 mL ROL vehicle +2 g/kg PARA
IV	8	PARA + NAC	140 mg/kg NAC +2 g/kg PARA
V	8	PARA + ROL 1.25 mg/kg	1.25 mg/kg ROL +2 g/kg PARA
VI	8	PARA + ROL 2.5 mg/kg	2.5 mg/kg ROL (ip) + 2 g/kg PARA
VII	8	PARA + ROL 5 mg/kg	5 mg/kg ROL +2 g/kg PARA

a PARA: Paracetamol; ROL: Rolipram; NAC: *N*-acetylcysteine; i.p.: Intraperitoneal.

**Figure 1:**
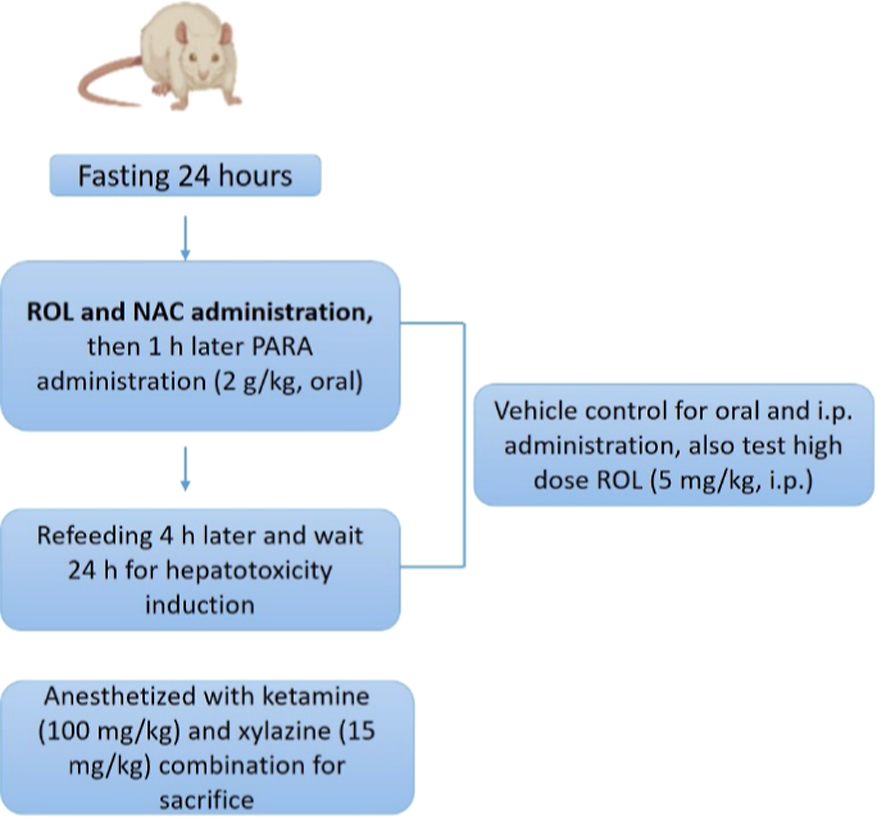
Summary of experimental procedure.

Prior to the experiment, the animals were fasted for 24 h. One hour after administration of ROL and NAC, PARA was administered at a dose of 2 g/kg and hepatotoxicity was induced by waiting 24 h postadministration. The PARA dose used in this study was selected based on relevant literature.^19,20^ Four hours after PARA administration, all rats were given free access to food and water until the end of the experiment. The control group received the same volume of the vehicles used in the study, administered orally and intraperitoneally. To evaluate the potential toxicity of ROL, a high dose (5 mg/kg) of ROL was administered to a separate group of healthy animals. Xylazine (15 mg/kg) and ketamine (100 mg/kg) were used to anesthetize the animals for sacrifice at the end of the experiment (Figure 1). Blood and liver tissue samples were collected from all rats. The collected blood samples blood samples were centrifuged for 10 min at 4 °C and 4000 rpm to separate the serum. Samples of serum and liver tissue were kept at −80 °C for molecular and biochemical analyses. Liver tissue samples were fixed in 10% (v/v) formalin for histological examinations, and then they were embedded in paraffin for histopathological investigation.

### 2.4 Biochemical Analysis

#### 2.4.1 Serum and Liver Tissue Analysis

Serum ALT and AST analysis were performed at the Medical Biochemistry Laboratories of Atatürk University Faculty of Medicine.

Serum and liver tissue samples were analyzed for cAMP and PDE4D levels using the ELISA method, performed on the epoch spectrophotometer system (BioTek Instruments, USA). The liver tissues were homogenized in liquid nitrogen using a TissueLyser II grinding jar set (Qiagen, Germany). Approximately 100 mg of the ground tissue was homogenized in 1 mL of PBS homogenization buffer using the TissueLyser II in an Eppendorf tube. After that, the homogenates were centrifuged for 15 min at +4 °C at 12,000 rpm. The supernatants obtained were analyzed for cAMP and PDE4D levels. All steps of the ELISA procedure were carried out in accordance with the manufacturer’s protocols.

#### 2.4.2 Protein Determination

The Lowry technique was used to quantify the total protein concentration in liver tissue.^21^ Commercial protein standards included in the Total Protein Kit (Sigma Chemical Co., Munich, Germany) were used during this procedure.

#### 2.4.3 Liver GSH Levels

Sedlak and Lindsay’s method was used to quantify glutathione (GSH) levels.^22^ GSH concentrations were calculated and expressed as nmol/mg protein. All samples and standards were analyzed in triplicate, and the results were reported as mean values.

### 2.5 Molecular Analyses

#### 2.5.1 Total RNA Isolation

Total RNA was isolated from approximately 30 mg of liver tissue stored at −80 °C, which was grounded in liquid nitrogen and homogenized in RLT buffer using the TissueLyser II according to the RNeasy Mini Kit (Qiagen) protocol. Following 3 min centrifugation at 14000 rpm, the supernatants collected and mRNA was extracted from the supernatant using the RNeasy Mini Kit on the QIAcube system (Qiagen). The RNA purity and concentration were measured using a NanoDrop spectrophotometer (Epoch Take3, BioTek). The isolated mRNA samples were used for subsequent complementary DNA (cDNA) synthesis.

#### 2.5.2 Reverse Transcription Reaction and cDNA Synthesis

cDNA synthesis was performed using the High Capacity cDNA Reverse Transcription Kit (Applied Biosystems) and a Veriti 96-Well Thermal Cycler (Applied Biosystems), following the manufacturer’s protocol. The resulting cDNA was stored at −20 °C until further real-time PCR analysis.

#### 2.5.3 Quantification of mRNA Expression Levels

GRP78, CHOP, and IRE1 mRNA expression levels were quantified using synthesized cDNA on a StepOnePlus Real-Time PCR System (Applied Biosystems) with β-actin as the endogenous control. Reactions were performed in triplicate using NucleoGene qPCR Probe Master Mix (2×) according to the manufacturer’s instructions. Cycle threshold (*C*_*t*_) values were automatically calculated and converted to delta delta *C*_*t*_ (^ΔΔ^ *C*_*t*_) values by the instrument software. Relative expression levels were calculated using the 2^^−ΔΔCt^ method and expressed as fold changes compared to the control group.^23^

### 2.6 Histopathological Analyses

To preserve structural integrity for microscopic examination, liver tissue samples were preserved in 10% neutral-buffered formaldehyde for at least 72 h. Following fixation, samples were dehydrated through a graded ethanol series, embedded in paraffin, and sectioned into 3–5 μm thick slices using a Leica RM2125RT microtome. The sections were mounted on glass slides and stained with Mallory’s trichrome stain, modified by Crossman, for routine histological evaluation. Stained slides were examined under a Zeiss AXIO Scope. Specific histopathological parameters, including necrosis, hemorrhage, and mononuclear cell infiltration, were evaluated in detail. Lesions were scored semiquantitatively based on severity as follows: absent (−), mild (+), moderate (++), severe (+++), and very severe (++++). Images were edited using Adobe Photoshop CS6 for documentation purposes.

### 2.7 Statistical Analyses

Statistical analyses were conducted using the Statistical Package for the Social Sciences (SPSS, version 20.0; SPSS Inc., Chicago, IL, USA). Skewness and Kurtosis values for all numerical data were between −1.5 and +1.5 and they were assumed to have normal distribution.^24^ So numerical data were subjected to statistical analysis using one-way analysis of variance (ANOVA) followed by Tukey’s post hoc test for multiple comparisons. Results are expressed as mean ± standard deviation (SD). For nonparametric data (histopathological scorings) that did not comply with normal distribution statistical analyses were performed using the Kruskal–Wallis test with post hoc analysis (Games–Howell test). A p-value of <0.05 was considered statistically significant.

## 3 Results

### 3.1 Serum ALT and AST of the Study Groups

ALT and AST, established biomarkers of liver injury, were measured to evaluate PARA induced liver injury and the effects of ROL pretreatment. As shown in Figure 2, ALT (A) and AST (B) levels significantly increased in the PARA group compared to the healthy control (*p* < 0.001). ROL reduced both ALT and AST levels with increasing doses in the PARA + ROL 1.25, 2.5, and 5 groups (all *p* < 0.001 vs PARA). However, ALT levels in the PARA + ROL 1.25 group (*p* < 0.05) and AST levels in the PARA + ROL 1.25 (*p* < 0.001) and PARA + ROL 2.5 (*p* < 0.05) groups remained higher than those in the healthy control significantly.

**Figure 2:**
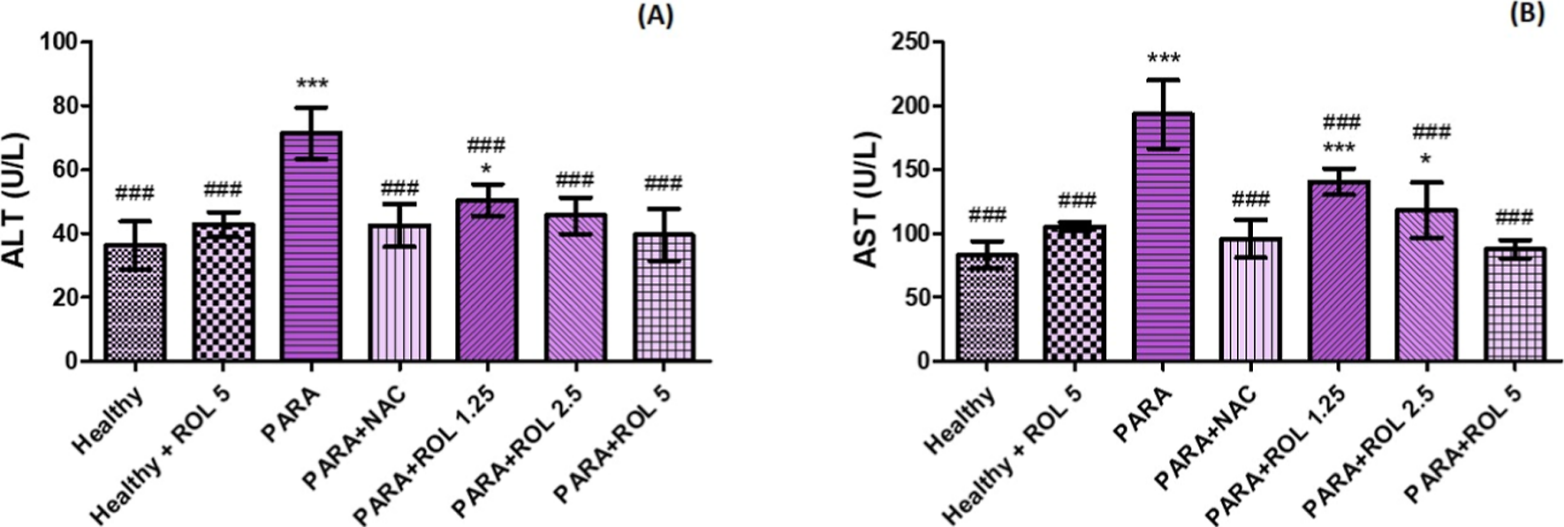
Serum ALT (A) and AST (B) levels in experimental groups. *p < 0.05; ***p < 0.001 vs healthy group; ###*p* < 0.001 PARA group Statistical analysis was conducted using one-way ANOVA followed by Tukey’s multiple comparison test. Data are expressed as mean ± SD PARA: Paracetamol, NAC: *N*-asetylcystein, ROL: Rolipram.

### 3.2 Hepatic GSH Levels

GSH levels were significantly decreased in the PARA group compared to the healthy group (*p* < 0.001), indicating oxidative stress. ROL administration led to increase in GSH levels dose dependently compared to the PARA group: PARA + ROL 1.25 (*p* > 0.05), PARA + ROL 2.5 (*p* < 0.01), and PARA + ROL 5 (*p* < 0.001), as shown in Figure 3.

**Figure 3:**
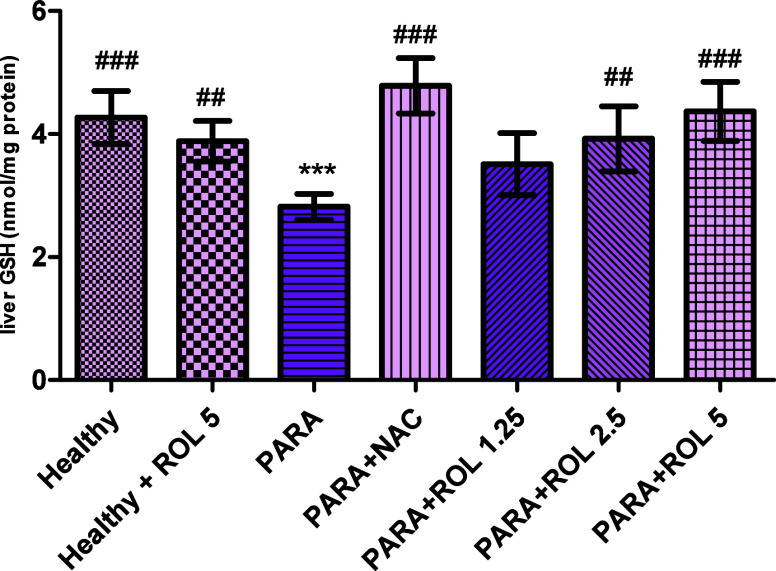
Hepatic GSH levels in experimental groups ***p < 0.001 vs healthy group; ##*p* < 0.01, ###*p* < 0.001 vs PARA group statistical analysis was conducted using one-way ANOVA followed by Tukey’s multiple comparison test. Data are expressed as mean ± SD PARA: Paracetamol, NAC: *N*-asetylcystein, ROL: Rolipram.

### 3.3 Serum and Hepatic cAMP and PDE4D Levels

Given that ROL is a selective PDE4 inhibitor, PDE4D levels were measured in both liver tissue and serum to evaluate its role in PARA-induced hepatotoxicity. Serum and liver tissue PDE4D levels showed a similar pattern. Both tissue and serum PDE4D levels significantly increased in the PARA group compared to controls (*p* < 0.05). ROL administration reduced PDE4D levels in a dose-dependent manner. At higher doses (2.5 and 5 mg/kg), levels fell below those of both the PARA and control groups: PARA + ROL 2.5 (*p* < 0.001), PARA + ROL 5 (*p* < 0.001), PARA + ROL 2.5 (*p* < 0.01) and PARA + ROL 5 (*p* < 0.001). Notably, liver tissue PDE4D levels in the PARA + ROL 2.5 and PARA + ROL 5 groups were even lower than those in the healthy control group (*p* < 0.05 and *p* < 0.01, respectively). Additionally, serum PDE4D levels in the PARA + ROL 2.5 and PARA + ROL 5 groups were significantly lower than those in the healthy group (*p* < 0.01 and *p* < 0.001, respectively). The healthy + ROL 5 group also showed significantly lower PDE4D levels than both control and PARA groups. These findings suggest that ROL effectively downregulates PDE4D expression in both liver and serum under toxic conditions (Figure 4A–D).

**Figure 4:**
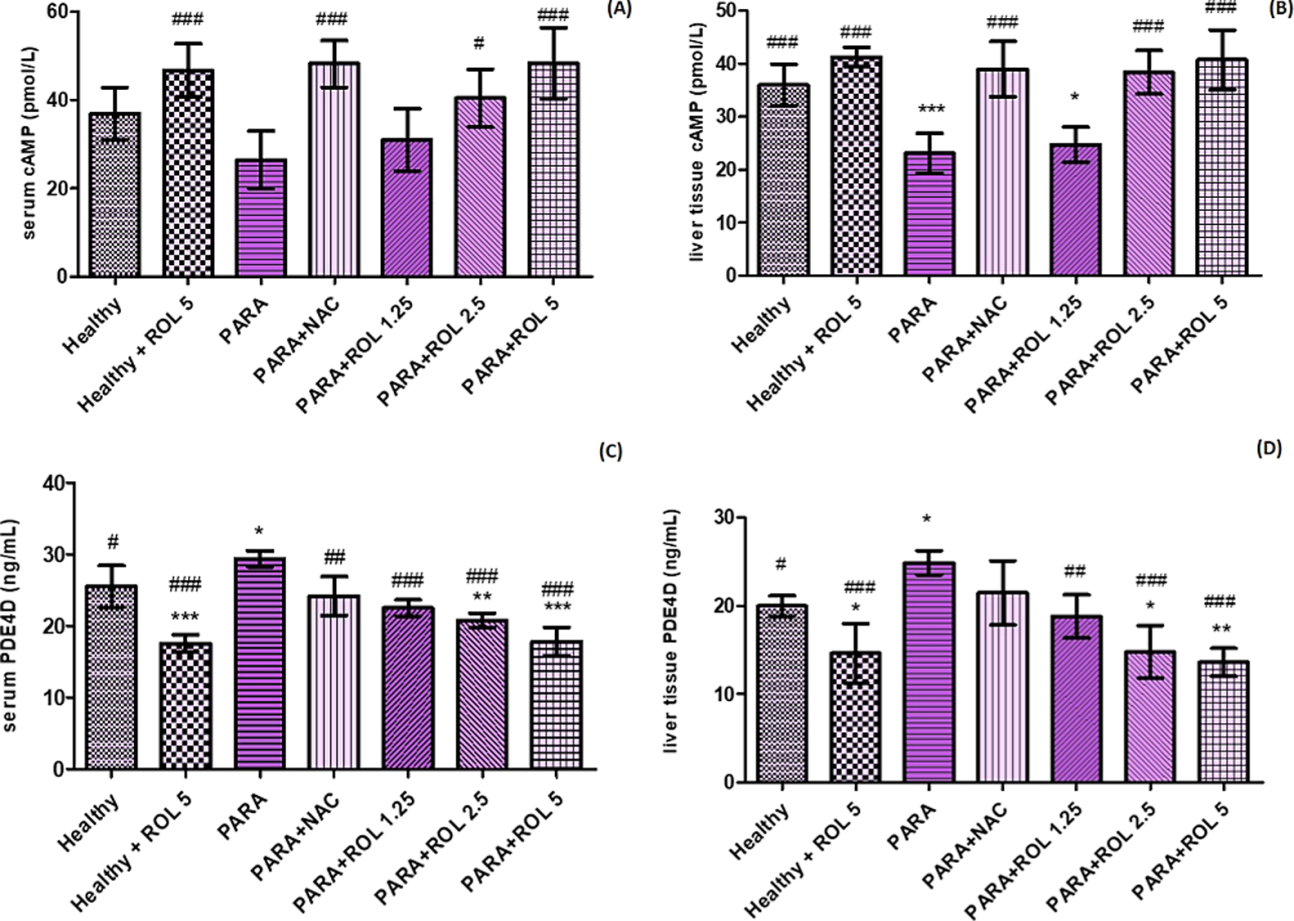
Serum/hepatic tissue cAMP and PDE4D levels *p < 0.05, **p < 0.01, ***p < 0.001 vs healthy group; #*p* < 0.05, ##*p* < 0.01, ###*p* < 0.001 vs PARA group Statistical analysis was conducted using one-way ANOVA followed by Tukey’s multiple comparison test. Data are expressed as mean ± SD PARA: paracetamol, NAC: *N*-asetylcystein, ROL: Rolipram.

As cAMP levels are expected to be influenced by PDE4 inhibition, serum and tissue were also evaluated in this study. Hepatic cAMP levels were significantly decreased in the PARA group compared to the healthy control (*p* < 0.001). ROL pretreatment led to a dose-dependent increase, with significant elevations in the PARA + ROL 2.5 and PARA + ROL 5 groups (*p* < 0.001 vs PARA). However, cAMP levels in PARA + ROL 1.25 remained lower than in the healthy group (*p* < 0.05). In serum, cAMP levels showed a nonsignificant decrease in the PARA group. ROL induced a dose-dependent increase, reaching significance in the PARA + ROL 2.5 (*p* < 0.05) and PARA + ROL 5 (*p* < 0.001) groups. Additionally, serum cAMP levels were significantly higher in the healthy + ROL 5 group compared to the healthy control (*p* < 0.001).

### 3.4 Molecular Findings

To investigate whether PDE4 inhibition modulates ERS in PARA induced liver injury, we measured hepatic mRNA expression levels of key ERS markers: GRP78, IRE1, and CHOP (Figure 5). PARA exposure significantly upregulated GRP78, IRE1, and CHOP mRNA levels compared to healthy controls (*p* < 0.001, for all). ROL pretreatment reduced the expression of these markers in a dose-dependent manner in a different significance levels compared to the PARA group. Notably, the highest dose (5 mg/kg) of rolipram led to a substantial decrease in GRP78 (*p* < 0.001), IRE1 (*p* < 0.001), and CHOP (*p* < 0.001) levels, approaching those of the control group (*p* < 0.001, for all). GRP78 expression is decreased in the PARA + ROL 1.25 group (*p* < 0.05) and the PARA + ROL 2.5 group (*p* < 0.01). IRE1 expression is decreased in both the PARA + ROL 1.25 and PARA + ROL 2.5 groups (all *p* < 0.001). CHOP expression is decreased in the PARA + ROL 1.25 group (*p* < 0.05) and the PARA + ROL 2.5 group (*p* < 0.001) when compared to the PARA group. However, at lower doses (1.25 and 2.5 mg/kg), mRNA levels of GRP78, IRE1, and CHOP remained significantly elevated compared to healthy controls. These results suggest that PDE4 inhibition by rolipram may attenuate PARA-induced ER stress, especially at higher doses.

**Figure 5:**
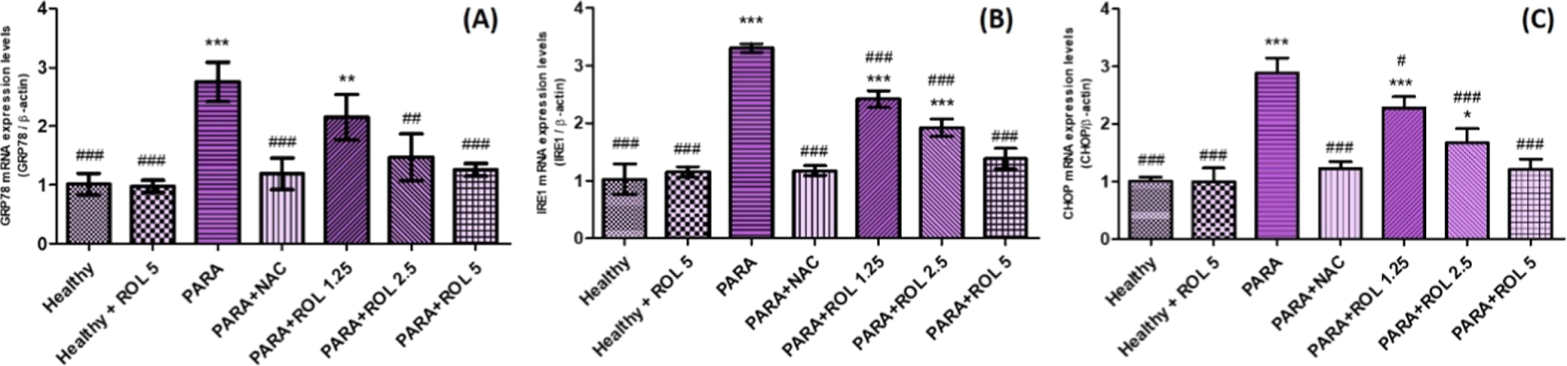
Hepatic GRP78, IRE1 and CHOP mRNA expression levels. *p < 0.05, **p < 0.01, ***p < 0.001 vs healthy control group; ##*p* < 0.01, ###*p* < 0.001 vs PARA group. Expression fold changes were calculated using the 2^^−ΔΔCt^ method relative to the healthy group. Statistical analysis was conducted using one-way ANOVA followed by Tukey’s multiple comparison test. Data are expressed as mean ± SD PARA: Paracetamol, NAC: *N*-acetylcysteine, ROL: Rolipram.

### 3.5 Histopathological Findings

Semiquantitative histopathological evaluations of liver tissues are presented in Table 2 with representative micrographs shown in Figure 6. The control group exhibited normal hepatic architecture without abnormalities (Figure 6A). PARA treatment caused severe damage, including hemorrhage, necrosis, mononuclear infiltration, vacuolization, and sinusoidal congestion (Figure 6B). The ROL-only group (5 mg/kg) maintained normal histology (Figure 6C). NAC post-treatment improved liver architecture, with reduced hemorrhage, inflammation, and necrosis (Figure 6D). PARA + ROL (1.25 mg/kg) showed mild improvement; necrosis and inflammation persisted to some extent (Figure 6E). PARA + ROL (2.5 mg/kg) exhibited more recovery, with reduced damage and improved hepatocyte morphology (Figure 5F). PARA + ROL (5 mg/kg) achieved the most pronounced recovery: nearly complete resolution of histopathological damage (Figure 6G).

**Table 2:** Semi-Quantitative Analysis of Hepatic Histopathological Alterations Among Groups^a^ ^,^ ^b^

groups	mononuclear cell infiltration	hemorrhage	necrosis
control	0.67***	0,33***	0.67***
ROL	1***	0.5***	0.67***
PARA	3.83	4	3.5
PARA + NAC	2.17	1.67**	1.33**
PARA + ROL1.25 mg/kg	3.17	3.33	3.33
PARA + ROL 2.5 mg/kg	3.17	3.17*	2.83
PARA + ROL 5 mg/kg	2.33	1.83**	1.83*

a Results were analyzed with the Kruskal–Wallis test (*p* < 0.05) with post hoc analysis (Games Howell; *p ≤ 0.05, **p ≤ 0.01, and ***p ≤ 0.001, when compared to paracetamol group).

b PARA: Paracetamol, NAC: *N*-asetylcystein, ROL: Rolipram.

**Figure 6:**
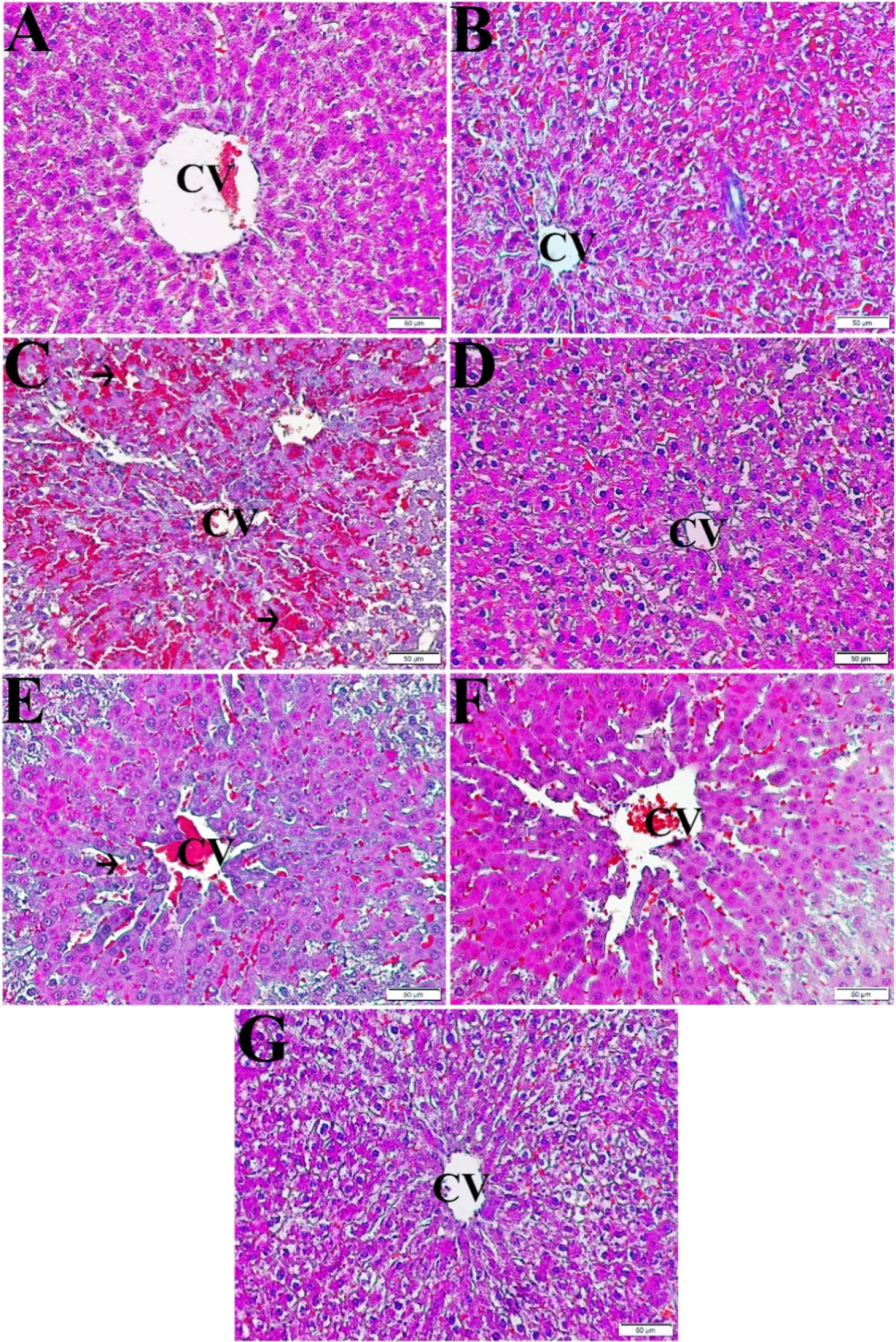
Light microscopic images of liver tissue sections (staining: Mallory’s trichrome stain modified by Crossman; Magnification ×200). A: healthy; B: healthy + ROL 5 mg/kg; C: PARA; D: PARA + NAC; E: PARA + ROL 1.25 mg/kg; F: PARA + ROL 2.5 mg/kg; G: PARA + ROL 5 mg/kg. CV: central vein; arrow: hemorrhagic areas.

## 4 Discussion

In this study, we demonstrated biochemically, histopathologically, and molecularly that ROL effectively ameliorates PARA induced liver injury in rats via inhibition of elevated PDE4 activity, restoration of decreased cAMP levels, and subsequent attenuation of ER stress.

First, serum liver injury biomarkers ALT and AST were measured, as these enzymes are released from damaged hepatocytes and reliably indicate hepatocellular injury, with elevated levels reported in both experimental and clinical PARA toxicity.^25^ In our study, 24 h after PARA administration, significant ALT and AST elevations in the PARA group confirmed hepatocellular damage, consistent with our previous models.^26,27^ ROL pretreatment reduced these enzymes in a dose-dependent manner, with high-dose ROL restoring levels comparable to NAC. Importantly, ROL administration alone did not elevate ALT or AST, confirming its hepatic safety and protective effect.

Second, hepatic GSH levels were assessed, as GSH depletion is a key feature of PARA toxicity due to NAPQI-mediated oxidative stress;^28^ therefore, it was selected as a primary indicator of oxidative injury in the present study. ROL has been shown to enhance intracellular GSH.^29^ Indeed, PARA reduced hepatic GSH, while ROL pretreatment dose-dependently restored it, suggesting antioxidative protection and preservation of hepatocyte membrane integrity. However, additional oxidative stress markers were not evaluated and should be included in future investigations for a more comprehensive assessment.

Previous studies have shown that phosphodiesterase inhibitors (pentoxifylline, galactozamine, sildenafil, aminophylline, vardenafil) reduce liver injury in hepatotoxic models.^30–34^ As seen in that studies, most of them focused on PDE5 inhibition. Since altered PDE4 activity may influence steatosis, inflammation, and fibrosis via cAMP, we investigated its role in PARA toxicity. We found that PARA increased PDE4 expression in serum and tissue, while ROL dose-dependently decreased it, correlating with cAMP restoration in serum and liver tissues.

The role of cAMP in liver disease has been documented.^8–10,17^ For example, glucagon-mediated cAMP elevation protects against PARA-induced injury.^8^ In another study, cAMP-mediated induction of IL-10 in liver cells was beneficial in LPS-induced inflammatory liver disease.^9^ In another study, it was shown that cAMP derivatives ameliorated carbon tetrachloride-induced cytoplasmic vacuolization in rat model not only by improving inflammation and lowering liver enzymes but also by inhibiting liver enzymes.^10^ Induction of decreased cAMP levels in the liver has also been shown to reduce alcohol-induced increased inflammation and steastosis in mice with ethanol-induced liver injury.^17^ In agreement, in our study, both serum and tissue cAMP levels decreased with PARA toxicity. However, this difference was not significant in serum cAMP levels, probably because the changes in the tissue were not immediately reflected in the serum levels. ROL dose-dependently restored cAMP, consistent with decreased PDE4 activity.

The cAMP/PKA signaling pathway has been reported to downregulates CYP2E1 activity.^11^ cAMP-mediated phosphorylation of CYP2E1 may reduce NAPQI formation, suggesting a potential mechanism by which ROL could attenuate PARA toxicity. A study evaluating the treatment of ethanol-induced hepatocyte death showed that cAMP modulated CYP2E1 and ADH activities, resulting in decreased ROS production and cell death.^35^ However, it should be noted that CYP2E1 activity and ROS production were not directly measured in our study. Therefore, these mechanistic interpretations are based on existing literature and should be considered speculative in the context of our findings.

Recent studies have demonstrated the role of ERS in PARA induced hepatotoxicity.^14,18,36^ A nonlethal dose of PARA results in the activation of ER stress markers, including ATF6 and CHOP. Additionally, Caspase-12, a caspase localized in the ER, is transiently activated.^18^ In a study evaluating the role of ERS in PARA induced hepatotoxicity using CHOP knockout mice, administration of toxic doses of PARA activated the ER stress pathway. The PERK-eIF2α-CHOP pathway was identified as the major signaling cascade of UPR activation signaling. Wild-type mice developed extensive hepatic necrosis, whereas CHOP knockout mice were partially protected from hepatotoxicity, showing improved survival rates and evidence of cell proliferation in necrotic areas. This suggests that upregulation of CHOP impairs hepatocyte survival by inhibiting regeneration.^14^ Cao et al. also demonstrated that PARA induced acute liver injury activates distinct ERS pathways, with increased expression of GRP78, PERK, and eIF2α observed in their study.^37^ Another study further confirmed activation of the PERK-eIF2α-ATF4 pathway in PARA-induced liver injury.^38^ Excessive doses of PARA were associated with elevated CHOP expression, while CHOP-deficient mice exhibited reduced toxicity and an enhanced regenerative response.^14^ In our study, we similarly observed that mRNA expression levels of GRP78, IRE1, and CHOP were elevated in the PARA toxicity group, once again supporting the role of ER stress in PARA induced liver damage. Administration of ROL at increasing doses with PARA significantly reduced the expression of ERS markers to the PARA toxicity group.

Histopathological analysis of liver tissues supported these biochemical and molecular findings and it was shown that increasing doses of ROL were effective in hepatocyte damage caused by PARA toxicity. While no pathological abnormalities were observed in healthy liver tissues, the PARA toxicity group exhibited severe hepatic injury characterized by extensive hemorrhage, prominent mononuclear cell infiltration, and widespread necrosis. These histopathological alterations were significantly ameliorated in ROL-treated groups, indicating the protective effect of ROL against PARA-induced hepatocellular damage.

Consistent with its established role, NAC significantly attenuated PARA-induced hepatotoxicity in our study, supporting the validity of the experimental model. The protective effects observed with ROL, particularly at higher doses, suggest that modulation of cAMP signaling may represent an alternative or complementary mechanism to NAC-mediated glutathione replenishment. Although NAC is the standard treatment for PARA toxicity, its efficacy is strongly dependent on early administration, and delayed treatment may reduce its protective capacity. Therefore, investigating agents that target additional mechanisms involved in hepatotoxicity remains of scientific and potential clinical interest. The dose dependent effects observed with ROL suggest that higher doses may provide greater hepatoprotective activity, although further studies are required to define the optimal therapeutic window.

This study has several limitations that should be considered. In order to assess sex-related variations in susceptibility to PARA-induced liver injury, only male rats were used. Second, the experimental design limited the capacity to evaluate the consequences of repeated or chronic exposures by focused on an acute toxicity model with a single large dosage of PARA. Third, although the rat model is commonly used to study hepatotoxicity, interspecies variations may limit the direct application of these results in clinical settings including humans. Fourth, the absence of a combination treatment group (NAC + ROL), which would be important to determine whether ROL provides additive or synergistic effects when used alongside standard therapy. And finally, it should be noted that ROL was administered prior to PARA exposure; therefore, the observed effects reflect a protective rather than a postexposure therapeutic action.

In conclusion, prior to this study, it remained unclear whether PARA toxicity alters hepatic PDE4 expression and cAMP levels, and whether impaired cAMP signaling plays a pathogenic role in PARA induced liver injury. As cAMP also functions as a regulator of ER stress, this study contributes novel insights to the literature. Our findings demonstrate that PDE4 inhibition by ROL mitigates PARA induced ER stress and liver injury, potentially via cAMP-mediated activation.

## Data Availability

The data that support the findings of this study are available on request from the corresponding author. The data are not publicly available due to privacy or ethical restrictions.
